# 分子靶向药物应用于非小细胞肺癌维持治疗的研究进展

**DOI:** 10.3779/j.issn.1009-3419.2010.11.14

**Published:** 2010-11-20

**Authors:** 利 单

**Affiliations:** 1 830011 乌鲁木齐，新疆医科大学附属肿瘤医院内一科 Department of Chemotherapy, Tumor Hospital Affiliated to Xinjiang Medical University, Urumqi 830011, China; 2 744000 平凉，甘肃省平凉市第二人民医院呼吸内科 Department of Respiratory Medicine, the Second People's Hospital of Pingliang, Pingliang 744000, China

在发达及发展中国家，肺癌仍是癌症死亡的重要原因^[1]^。其中，非小细胞肺癌（non-small cell lung cancer, NSCLC）占80%-85%，并且70%以上NSCLC患者临床确诊时处于复发或转移性的晚期阶段，失去手术根治机会，因此内科治疗在NSCLC治疗过程中占据重要地位。对于大多数晚期NSCLC患者，一线标准化疗为4个-6个周期的铂类联合第三代细胞毒性药物，其有效率为20%-30%，中位生存期（median survival time, MST）为8个月-10个月，疾病进展时间（time to progression, TTP）为3个月-5个月^[2]^。一线标准化疗方案已经取得共识且在一定程度上可以延长NSCLC患者的生存期，但疗效已达到平台，从而使分子靶向药物脱颖而出，成为NSCLC内科治疗的新热点^[3]^。NSCLC患者在经过一线标准化疗并获得客观缓解或疾病稳定后，如何应用耐受性好、无毒性积累及疗效佳的药物乘胜追击进行维持治疗，以巩固疗效并从治疗中获益，成为目前NSCLC治疗过程中的焦点问题。分子靶向药物因具有较好的安全性和耐受性，作为晚期NSCLC维持治疗用药着实令人期待。本文将对分子靶向药物在晚期NSCLC维持治疗中的研究进展进行综述。

## 1 概述

### 1.1 NSCLC分子靶向治疗概述

癌症的分子靶向治疗（molecular targeted therapy）是指以癌症相关分子作为靶点，将药物、抗体等有效成分靶向定位于癌细胞及相关成分，从而达到治疗癌症的目的^[4]^。分子靶向治疗具有高效、不良反应较低的特点，因而越来越被广大肿瘤学研究者所认同。NSCLC分子靶向治疗常用的治疗靶点有细胞受体、信号传导和抗血管生成等^[5]^。目前晚期NSCLC药物治疗正从单纯细胞毒性药物转化到分子靶向治疗的时代^[6]^。

### 1.2 维持治疗的概念

维持治疗（maintenance therapy）是指患者完成初始化疗既定的化疗周期数并达到最大肿瘤控制效应后再继续接受药物治疗^[7]^。用于NSCLC维持治疗的药物可以是诱导化疗方案中的一种药物，也可以是另外一种相对低毒的非交叉耐药的药物，所用药物的剂量较小。维持治疗的时间为直至NSCLC病情进展或因患者无法耐受毒副反应而被迫中止^[8]^。目前尽管对于NSCLC维持治疗尚存诸多争议，但其有可能延缓NSCLC的复发，延长患者生存期，从而逐渐得到重视^[9]^，尤其随着分子靶向药物应用于临床，其特异性靶向作用以及轻微的不良反应使得它在NSCLC维持治疗中更具吸引力。

### 1.3 NSCLC维持治疗的基本原理及现状

肺癌维持治疗的理论基础最早由外国学者Goldie等^[10]^提出，即尽早使用非交叉抑制药物可以在耐药产生前增加杀伤肿瘤细胞的效能，使治疗效果最优。NSCLC患者体内对于前期化疗耐药的细胞数量会随着疾病发展不断增加和累积，尽早使用无交叉耐药的化疗药物可以在耐药前杀灭更多癌细胞^[11]^。所以选取有效的药物或方案作为前期NSCLC化疗后的维持治疗，可以使该疗效达到最佳。近年来，NSCLC维持治疗为临床提供了新的思路，成为肺癌学术界的研究焦点之一，其中临床研究较多的药物是吉西他滨、多西他赛和培美曲赛^[12-14]^，上述三药已被美国食品药品管理局（Food and Drug Administration, FDA）批准应用于NSCLC维持治疗。2010版NCCN肿瘤学临床实践指南^[15]^推荐且国内批准的NSCLC维持治疗方案如下：在含铂两药联合方案一线化疗4个-6个周期之后可开始多西他赛维持治疗。随着对肿瘤发病机制的进一步认识及分子生物学的发展，人们逐渐认识到，使用分子靶向药物维持治疗，在保证前期治疗效果的同时也减少了毒性累加及相关副作用，其在NSCLC维持治疗中的地位举足轻重。

## 2 分子靶向药物与NSCLC维持治疗

随着传统的化疗药物联合治疗方案已经进入平台期，分子靶向治疗的地位逐渐升高，使得分子靶向药物成为NSCLC治疗中的一支生力军，尤其是新近公布的一系列NSCLC靶向治疗的临床研究结果，显示分子靶向药物应用于NSCLC维持治疗中作为一种延长患者生存期及提高患者生存质量的手段，拥有鼓舞人心的前景。

### 2.1 表皮生长因子受体（epidermal growth factor receptor, EGFR）抑制剂

EGFR是跨膜糖蛋白受体Erb-B家族成员之一，其主要过度表达于NSCLC中，以腺癌最常见（40.0%）。当配体与受体胞外区结合后，EGFR活化形成同源或异源二聚体形式，导致胞内TK区被激活，使酪氨酸自身磷酸化，引起下游信号通路活化，导致细胞异常增殖和分化、血管生成增加并抑制肿瘤细胞凋亡^[16]^。EGFR抑制剂进入临床试验和应用阶段的主要是小分子酪氨酸激酶抑制剂（tyrosine kinase inhibitor, TKI）和人源化单克隆抗体，前者包括有吉非替尼和厄洛替尼，后者的典型代表为西妥昔单抗^[17]^。

吉非替尼（Gefitinib，ZD1839，Iressa，易瑞沙）主要是阻断了EGF、TGFα与EGFR的结合，阻止EGFR同源和异源二聚体的形成。早在2003年，就有学者^[18]^提出诱导化疗后给予吉非替尼维持治疗，从理论上有可能提高和巩固化疗的疗效，并进一步提高吉非替尼的疗效。随后2007年，美国临床肿瘤学会（American Society of Clinical Oncology, ASCO）年会公布了有关SWOG 0023研究^[19]^结果：该研究对Ⅲ期NSCLC患者放疗同步联合多西紫杉醇化疗后以吉非替尼与安慰剂作维持治疗进行比较，吉非替尼维持治疗组总生存期（overall survival, OS）为23个月，比安慰剂组35个月的OS反而明显减少（*P* =0.013），吉非替尼维持组不良反应主要为3/4级皮疹（7%）、腹泻（7%），该试验失败原因有待进一步研究。2008年，日本学者Hida等^[20]^报道的WJTOG 0203研究显示：对603例Ⅲb/Ⅳ期亚洲NSCLC患者进行研究，患者随机分为两组，分别接受3个-6个周期以铂类为主的化疗（298例）或3周期含铂两药联合化疗后吉非替尼维持治疗（300例），两者比较发现，使用吉非替尼进行维持治疗的患者无进展生存期（progression free survival, PFS）显著延长（4.6个月*vs* 4.2个月，*P* < 0.001），两组MST与OS无统计学差异（*P*=0.10），但是进一步比较发现维持治疗组病理分型为腺癌的患者OS明显延长（15.4个月，*P*=0.03）。上述资料表明吉非替尼作为NSCLC维持治疗用药可能使患者获益，尤其是亚洲腺癌患者。

厄洛替尼（Erlotinib，OSI2774，Tarceva，特罗凯）是另外一种高效、口服、高特异性、可逆的EGFR酪氨酸激酶抑制剂，对HERI/EGFR有高度的选择性抑制作用，主要通过抑制EGFR酪氨酸激酶磷酸化反应，从而抑制信号转导达到抑制癌细胞增殖作用。2005年Herbst等^[21]^报道的一项临床Ⅲ期试验是有关厄洛替尼在NSCLC维持治疗中的有效性，该试验中，861例NSCLC患者在经过铂类为主的常规化疗方案治疗后病情得到控制且预计生存期超过4个月，随机分为两组，其中厄洛替尼维持治疗组408例，安慰剂对照组453例，两组的OS分别为13.6个月和12.2个月（*P*=0.04）。2008年，ASCO年会报道了一项FAST-ACT Ⅱ期临床试验^[22]^：对154例（94%亚洲人）NSCLC初治患者予以4个周期GC方案化疗与厄洛替尼交替治疗，患者病情稳定后再给予厄洛替尼或安慰剂（两组腺癌患者均占2/3）维持治疗，结果显示厄洛替尼组的PFS显著延长（7.2个月*vs* 5.5个月，*P*=0.005），OS尚未统计。Cappuzzo等^[23]^公布了人们期待已久的SATURN研究结果，该试验共纳入1 949例一线化疗后未进展的晚期NSCLC患者，随机分组后给予厄洛替尼或安慰剂维持治疗直至疾病进展，结果显示厄洛替尼组的PFS较安慰剂组延长（12.3周*vs* 11.0周，*P* < 0.000 1），亚组分析显示不同性别、不同病理类型、不同人种、不同吸烟状况、EGFR野生型和突变型的患者都可以从厄洛替尼维持治疗中获益。2010年NCCN指南第一版推荐：在含铂类两药联合方案一线化疗4个-6个周期后可开始使用厄洛替尼维持治疗，但国内尚未批准用于NSCLC维持治疗。

西妥昔单抗（Cetuximab，C225，Erbitux，爱必妥）是人源化人鼠嵌合性抗EGFR单克隆抗体，在EGFR胞外结合区，与自然配体竞争受体结合位点，阻断配体与EGFR结合，从而抑制配体诱导的酪氨酸激酶活化，还能抑制血管生成，从而抑制癌细胞增殖与转移。2008年Pirker等^[24]^利用西妥昔单抗进行NSCLC维持治疗研究，相对于化疗，利用西妥昔单抗进行维持治疗的患者的OS有一定的延长，但由于仅使用西妥昔单抗治疗4周，因此该试验结果尚不足以采信。随后2009年，Gandara等^[25]^报道美国西南肿瘤协作组（Southwest Oncology Group, SWOG）开展的一项Ⅲ期临床试验，纳入110例NSCLC患者经过前期诱导治疗后，继续采用西妥昔单抗联合贝伐珠单抗进行维持治疗，疾病控制率为77%，PFS为7个月，OS为14个月，1年生存率为57%。2010版NCCN指南推荐：在4个-6个周期顺铂+长春瑞滨联合西妥昔单抗方案治疗NSCLC后可使用西妥昔单抗继续维持治疗，但国内尚未批准。

### 2.2 VEGF抑制剂

近年血管内皮生长因子（vascular endothelial growth factor, VEGF）抑制剂成为肿瘤靶向治疗的研究热点，血管生成在肿瘤的发生、发展和转移中都起到非常重要的作用，可以通过抑制血管生成来达到控制肿瘤的目的。VEGF是目前发现的参与肿瘤血管形成作用最强和最特异的生长因子，故VEGF及其受体（VEGFR）被认为是最有前途的抗肿瘤血管生成靶点。目前，VEGF抑制剂应用于NSCLC维持治疗研究最多的是贝伐珠单抗。

贝伐珠单抗（Bevacizumab, Avastin）是一种重组的人类单克隆IgG抗体，通过与VEGFR特异性结合，阻断VEGF的生物学效应，进而抑制肿瘤血管生成。ECOG4599^[26]^与AVAiL^[27]^两项研究中，对于经过治疗疾病得到控制的NSCLC患者研究组均给予贝伐珠单抗单药维持，结果提示联合贝伐珠单抗作为NSCLC维持治疗在PFS方面具有明显的优势，但是OS没有明显改善，且贝伐珠单抗在治疗鳞癌过程中曾出现大咯血及消化道穿孔大出血导致患者死亡等不良事件，其试验安全性着实令人担忧。2007年，TC方案联合贝伐珠单抗作为晚期非鳞癌NSCLC一线治疗的推荐方案写入指南。根据2009年ASCO年会会议报道，Miller等^[28]^进行了一项Ⅲ期临床试验（ATLAS研究），旨在比较NSCLC患者经一线含铂化疗+贝伐珠单抗治疗病情得到控制后，继续使用贝伐珠单抗单药维持治疗与贝伐珠单抗+厄洛替尼维持治疗的疗效，结果显示贝伐珠单抗+厄洛替尼组PFS为4.8个月，贝伐珠单抗组为3.7个月（*P*=0.001 2）。在上述几项研究中使用贝伐珠单抗的主要不良反应为皮疹和腹泻。Fred Hutchinson肿瘤研究中心^[29]^正在进行贝伐珠单抗联合伊马替尼的NSCLC维持治疗临床试验，其结果令人期待。2010年NCCN指南推荐：在4个-6个周期含铂类两药化疗联合贝伐珠单抗之后可使用贝伐珠单抗继续维持治疗，但国内尚未用于NSCLC维持治疗。综上所述，以贝伐珠单抗为代表的大分子靶向药物在NSCLC维持治疗中的地位得到初步肯定。

## 3 结语

根据现有的临床研究资料，证实NSCLC患者在接受标准的一线化疗病情得到控制后，利用分子靶向药物作为维持治疗能带来一定临床获益，主要表现为PFS的延长，但是仅改善PFS的意义有限，除非能同时控制肿瘤症状、减轻并发症或改善生活质量。在分子靶向药物作为NSCLC维持治疗的研究中，仍存在诸如其对OS的影响、患者纳入标准的制定、维持治疗方案与持续时间的统一、存在一定的不良反应以及治疗费用昂贵等问题，这需要多中心、大样本、随机、对照临床试验数据为分子靶向药物维持治疗NSCLC提供依据，在此基础上我们是否可以应用个体化的思路来为其找到更为广阔的出路。总而言之，分子靶向药物维持治疗NSCLC前景光明，值得期待。
